# Androgen Receptor Signaling Induces Cisplatin Resistance via Down-Regulating GULP1 Expression in Bladder Cancer

**DOI:** 10.3390/ijms221810030

**Published:** 2021-09-17

**Authors:** Yuki Teramoto, Guiyang Jiang, Takuro Goto, Taichi Mizushima, Yujiro Nagata, George J. Netto, Hiroshi Miyamoto

**Affiliations:** 1 Department of Pathology & Laboratory Medicine, University of Rochester Medical Center, Rochester, NY 14642, USA; yuki_teramoto@urmc.rochester.edu (Y.T.); gyjiang@cmu.edu.cn (G.J.); takuro@uro.med.tohoku.ac.jp (T.G.); mizu123shima@gmail.com (T.M.); yujiro-nagata@med.uoeh-u.ac.jp (Y.N.); 2 James P. Wilmot Cancer Institute, University of Rochester Medical Center, Rochester, NY 14642, USA; 3 Department of Pathology, China Medical University, Shenyang 110122, China; 4 Department of Pathology, Johns Hopkins University School of Medicine, Baltimore, MD 21287, USA; gnetto@uabmc.edu; 5 James Buchanan Brady Urological Institute, Johns Hopkins University School of Medicine, Baltimore, MD 21287, USA; 6 Department of Pathology, University of Alabama at Birmingham, Birmingham, AL 35249, USA; 7 Department of Urology, University of Rochester Medical Center, Rochester, NY 14642, USA

**Keywords:** androgen receptor, chemoresistance, cisplatin, immunohistochemistry, urothelial cancer

## Abstract

The underlying molecular mechanisms of resistance to cisplatin-based systemic chemotherapy in bladder cancer patients remain to be elucidated, while the link between androgen receptor (AR) activity and chemosensitivity in urothelial cancer has been implicated. Our DNA microarray analysis in control vs. AR knockdown bladder cancer lines identified GULP1 as a potential target of AR signaling. We herein determined the relationship between AR activity and GULP1 expression in bladder cancer cells and then assessed the functional role of GULP1 in cisplatin sensitivity. Androgen treatment in AR-positive cells or AR overexpression in AR-negative cells considerably reduced the levels of GULP1 expression. Chromatin immunoprecipitation further showed direct interaction of AR with the promoter region of GULP1. Meanwhile, GULP1 knockdown sublines were significantly more resistant to cisplatin treatment compared with respective controls. GULP1 knockdown also resulted in a significant decrease in apoptosis, as well as a significant increase in G2/M phases, when treated with cisplatin. In addition, GULP1 was immunoreactive in 74% of muscle-invasive bladder cancers from patients who had subsequently undergone neoadjuvant chemotherapy, including 53% of responders showing moderate (2+)/strong (3+) expression vs. 23% of non-responders showing 2+/3+ expression (*P* = 0.044). These findings indicate that GULP1 represents a key downstream effector of AR signaling in enhancing sensitivity to cisplatin treatment.

## 1. Introduction

Urinary bladder cancer, mostly urothelial carcinoma, has been one of the most commonly diagnosed malignancies, especially in men [1,2]. Moreover, the number of newly diagnosed bladder cancer cases and related deaths throughout the world has even risen from 429,800 and 165,100 estimated in 2010 [1] to 549,393 and 199,922 reported in 2018 [2], respectively. Clinically, there are two distinct forms of bladder cancer, non-muscle-invasive and muscle-invasive diseases, and the latter is often associated with metastatic disease where the overall 5-year survival rate remains low (i.e., 6.4% [3]). Urothelial carcinoma also occurs in the upper urinary tract, which is often (i.e., 60% [4]) invasive at the time of initial diagnosis.

Although new therapeutic options, such as immune checkpoint blockade, have become available, cisplatin (CDDP)-based combination chemotherapy remains the first-line standard for locally advanced or metastatic urothelial carcinoma [5,6]. However, a considerable number of patients fail to respond to such systemic chemotherapy. Specifically, the response rate to a CDDP-containing regimen has been reported to be 40–60% in bladder cancer patients undergoing neoadjuvant chemotherapy followed by radical cystectomy [7,8]. Accordingly, the development of strategies for not only overcoming chemoresistance but also predicting chemosensitivity constitutes a goal with critical clinical implications.

The underlying mechanisms for CDDP resistance are still to be established, while it has been suggested that multiple molecular pathways, including extracellular signal-regulated kinases (ERKs) and PTEN, are involved in the modulation of the cytotoxic and anti-proliferative activity of CDDP [9,10]. Meanwhile, androgen receptor (AR) signaling has been implicated in the induction of urothelial tumorigenesis [11], which may clearly explain the male dominance in the incidence of bladder cancer. More interestingly, AR activation has been linked to resistance to conventional non-surgical treatments for bladder cancer [12], including CDDP therapy [13,14,15], as well as intravesical bacillus Calmette-Guѐrin immunotherapy [16] and radiotherapy [17]. Nonetheless, it needs to be further elucidated how androgen-mediated AR signals control chemosensitivity in urothelial cancer. In the present study, we aim to investigate whether GULP1, an adaptor protein known to facilitate phagocytosis [18], represents a downstream effector of AR and thereby modulates CDDP sensitivity in bladder cancer.

## 2. Results

### 2.1. Associations between AR and GULP1 Expression

We recently employed DNA microarray analysis in control AR-positive UMUC3 versus a UMUC3 subline stably expressing AR-shRNA [16]. Of those expressed at absolutely high levels, several candidate genes were examined if their expression was not only up-regulated in AR-knockdown cells but also down-regulated in CDDP-resistant cells. Indeed, a quantitative PCR confirmed the significant increase or decrease in the levels of GULP1 expression in AR-knockdown or CDDP-resistant subline, respectively, compared with control cells. We thus decided to further investigate the functions of GULP1 whose role in neoplastic diseases was largely unknown.

**Figure 1:**
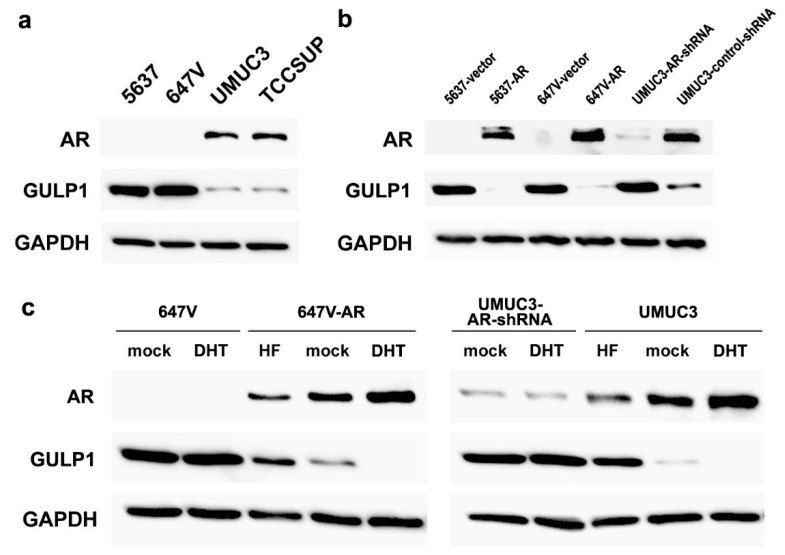
Relationship between AR and GULP1 expression in bladder cancer cells. (**a**) Western blotting of AR and GULP1 in 5637, 647V, UMUC3, and TCCSUP. (**b**) Western blotting of AR and GULP1 in 5637-vector vs. 5637-AR, 647V-vector vs. 647V-AR, and UMUC3-AR-shRNA vs. UMUC3-control-shRNA. (**c**) Western blotting of AR and GULP1 in 647V/647V-AR or UMUC3-AR-shRNA/UMUC3, cultured for 24 h with ethanol (mock), 10 nM DHT, or 5 µM HF. GAPDH served as a loading control.

We first examined the expression of GULP1 in four human bladder cancer lines that were known to be AR-negative (i.e., 5637, 647V) or AR-positive (i.e., UMUC3, TCCSUP) [19]. Western blot detected GULP1 signals in all of these cell lines, and the levels were higher in AR-negative cells than in AR-positive cells (Figure 1a). We then compared the levels of GULP1 expression in AR-negative/knockdown versus AR-positive/overexpression sublines. Consistent with the DNA microarray data [16], GULP1 expression was down-regulated in cells highly expressing AR (Figure 1b). We further assessed the effects of androgen (i.e., dihydrotestosterone (DHT)) and anti-androgen (i.e., hydroxyflutamide (HF)) on GULP1 expression. DHT treatment (vs. mock treatment) did not significantly change the levels of GULP1 expression in AR-negative 647V or UMUC3-AR-shRNA cells (Figure 1c). However, in AR-expressing cells, DHT and HF considerably reduced and induced, respectively, GULP1 expression over mock treatment. Thus, the expression of GULP1 was inversely related to the expression/activity of AR in bladder cancer cells.

**Figure 2:**
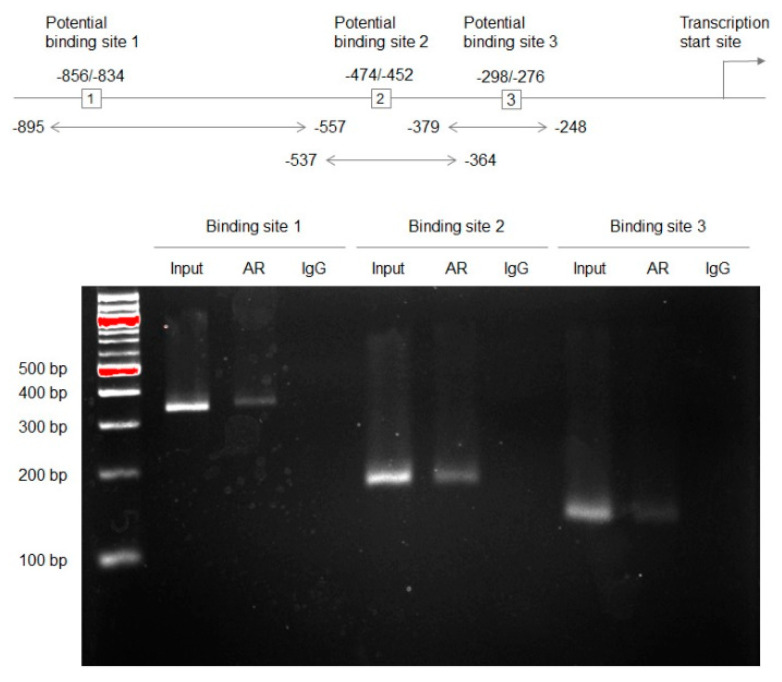
Binding of AR to the GULP1 promoter in bladder cancer cells. There are three putative binding sites in the GULP1 promoter region. A ChIP assay using UMUC3 cell lysates immunoprecipitated with an anti-AR antibody or mouse IgG as a negative control. The DNA fragments were PCR amplified with sets of GULP1 promoter-specific primers, and the PCR products (i.e., 338 bp for binding site 1, 173 bp for binding site 2, 131 bp for binding site 3) were electrophoresed on 1% agarose gel. A fraction of the mixture of protein-DNA complex (i.e., 1% of total cross-linked, reserved chromatin prior to immunoprecipitation) was used as “input” DNA.

A bioinformatics-driven search identified three potential AR binding sites in the GULP1 promoter region. We therefore investigated whether AR could regulate the expression of GULP1, using a chromatin immunoprecipitation (ChIP) assay (Figure 2). DNA fragments from UMUC3 cells immunoprecipitated with an anti-AR antibody or normal immunoglobulin G (IgG) were amplified by PCR with sets of GULP1 promoter-specific primers. The PCR products for all three putative binding sites could be visualized from those precipitated by the AR antibody, but not control precipitations, indicating interactions of AR with the GULP1 promoter.

### 2.2. Associations between GULP1 Expression and CDDP Sensitivity

**Figure 3:**
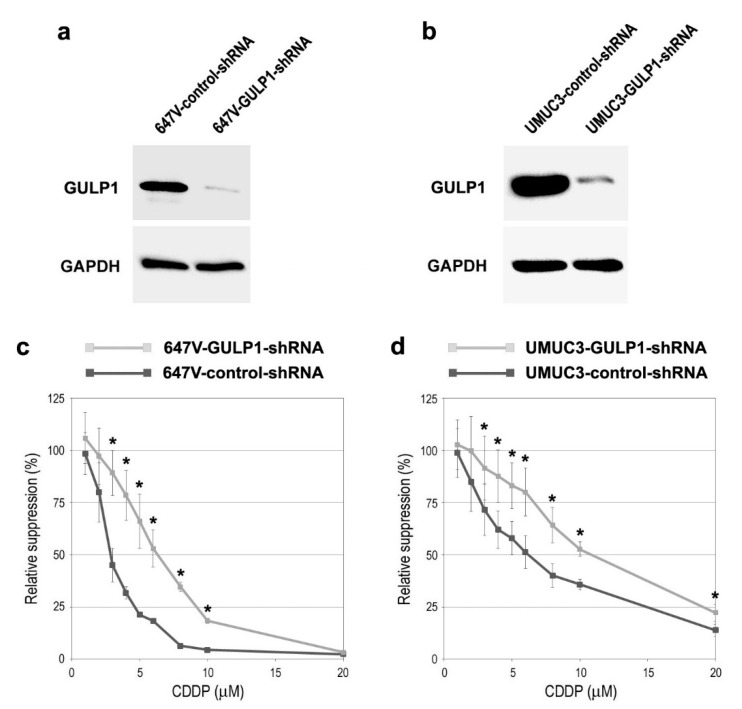
Effects of GULP1 knockdown on CDDP cytotoxicity in bladder cancer cells. Western blotting of GULP1 in 647V-control-shRNA vs. 647V-GULP1-shRNA sublines (**a**) or UMUC3-control-shRNA vs. UMUC3-GULP1-shRNA sublines (**b**). GAPDH served as a loading control. An MTT assay in 647V-control-shRNA vs. 647V-GULP1-shRNA sublines (**c**) or UMUC3-control-shRNA vs. UMUC3-GULP1-shRNA sublines (**d**) cultured for 72 h in the presence of various concentrations (0–20 µM) of CDDP. Cell viability is presented relative to that of each subline without CDDP treatment. Each value represents the mean (±SD) from a total of 6 determinants. * *p* < 0.05 (vs. control-shRNA).

Next, we assessed the impact of GULP1 expression on sensitivity to CDDP treatment in bladder cancer cells. To compare the cytotoxic effects of CDDP in GULP1-positive versus GULP1-negative cells, we silenced GULP1. As expected, the levels of GULP1 were substantially lower in 647V (Figure 3a) and UMUC3 (Figure 3b) sublines stably expressing GULP1-shRNA than in respective control-shRNA-expressing sublines. The MTT assay performed in these sublines treated with various doses of CDDP covering its pharmacological concentrations (i.e., 1.3–8.4 µM [20]) then showed that 647V-GULP1-shRNA (Figure 3c) and UMUC3-GULP1-shRNA (Figure 3d) were significantly more resistant to CDDP (i.e., 3–10 µM), compared with respective control sublines. The 50% inhibitory concentrations (IC50s) were: 3.0 µM (in 647V-control-shRNA) vs. 6.3 µM (in 647V-GULP1-shRNA); and 6.1 µM (in UMUC3-control-shRNA) vs. 10.8 µM (in UMUC3-GULP1-shRNA).

### 2.3. Role of GULP1 in Cell Growth

**Figure 4:**
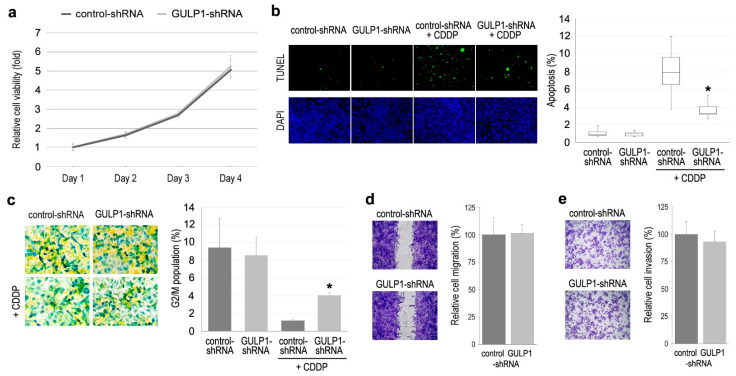
Effects of GULP1 knockdown on the growth of bladder cancer cells. (**a**) MTT assay in 647V-control-shRNA vs. 647V-GULP1-shRNA sublines cultured for 1–4 days. Cell viability is presented relative to that of the control subline at day 1. Each value represents the mean (±SD) from a total of 6 determinants. (**b**) TUNEL assay in 647V-control-shRNA vs. 647V-GULP1-shRNA sublines in the absence or presence of 5 µM CDDP cultured for 72 h. Apoptosis counted as a percentage of at least 500 cells is presented relative to that of the control subline. Each value represents the median (±SE) from a total of 16 determinants. (**c**) Cell cycle phase analysis in 647V-control-shRNA vs. 647V-GULP1-shRNA sublines in the absence or presence of 5 µM CDDP cultured for 72 h. Color changes are associated with cells in G1 (yellow), S (light green), G2 (dark green), and M (intense blue) phases (original magnification: 100×). Proportion of G2/M counted as a percentage represents the mean (+SD). (**d**) Wound-healing assay in 647V-control-shRNA vs. 647V-GULP1-shRNA sublines gently scratched and cultured for 24 h. Cell migration determined by the rate of cells filling the wound area is presented relative to that of the control subline (original magnification: 40×). Each value represents the mean (+SD) from a total of 10 determinants. (**e**) Transwell invasion assay in 647V-control-shRNA vs. 647V-GULP1-shRNA sublines. Cell invasion determined by counting the number of invaded cells in the lower chamber under a microscope is presented relative to that of the control subline (original magnification: 40×). Each value represents the mean (+SD) from a total of 10 determinants. * *p* < 0.001 (vs. control-shRNA).

Using control-shRNA vs. GULP1-shRNA sublines, we assessed the role of GULP1 in the cell proliferation (via MTT assay (Figure 4a)), apoptosis (via TUNEL assay (Figure 4b)), cell cycle (Figure 4c), cell migration (via wound-healing assay (Figure 4d)), and cell invasion (via transwell invasion assay (Figure 4e)). In these assays without CDDP treatment, there were no significant differences between the two sublines. However, in the presence of CDDP, GULP1 knockdown resulted in a significant decrease in CDDP-induced apoptosis (Figure 4b), as well as a significant increase in CDDP-reduced G2/M population (Figure 4c).

### 2.4. Expression of GULP1 in Bladder Cancer Specimens

**Figure 5:**
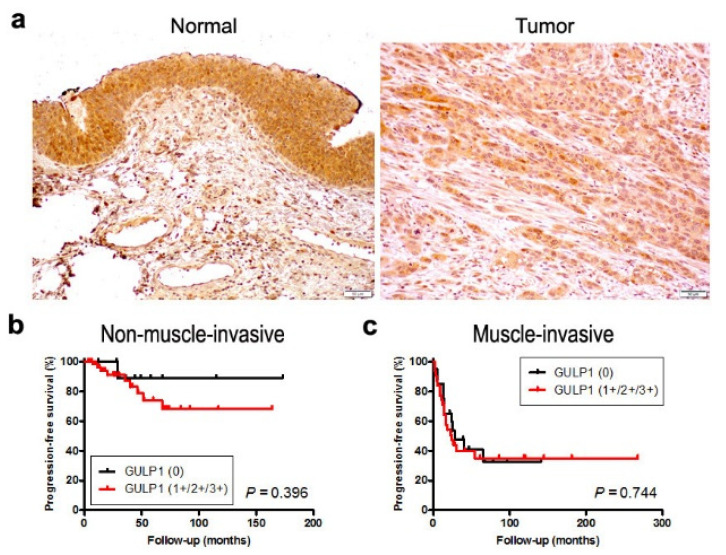
Immunohistochemistry of GULP1 in surgical specimens. (**a**) Expression of GULP1 in non-neoplastic urothelium and urothelial tumor (original magnification: 200×). Immunoreactivity is seen mainly in the cytoplasm of urothelial cells. (**b**) Kaplan–Meier curves for progression-free survival in patients with GULP1-negative (*n* = 13) vs. GULP1-positive (*n* = 65) non-muscle-invasive tumors. (**c**) Kaplan–Meier curves for progression-free survival in patients with GULP1-negative (*n* = 20) vs. GULP1-positive (*n* = 31) muscle-invasive tumors.

We stained immunohistochemically for GULP1 in two separate sets of bladder tissue microarrays (TMAs). Positive signals were detected predominantly in the cytoplasm of non-neoplastic and neoplastic epithelial cells (Figure 5a).

In the first set of TMA consisting of 129 cases of urothelial neoplasms and corresponding 89 non-neoplastic normal-appearing urothelial tissues, GULP1 was positive in 80 (90%) of non-neoplastic and 96 (74%) of neoplastic specimens (Table 1). Thus, the rate of GULP1 positivity was significantly lower in tumors than in non-neoplastic tissues. In addition, GULP1 expression was marginally or significantly down-regulated in high-grade (68%) or muscle-invasive (61%) tumors compared with lower grade (84%) or non-muscle-invasive (83%) tumors, respectively. There was no statistically significant difference in GULP1 positivity between muscle-invasive cases showing pN0 (64%) versus pN+ (46%). We then performed a Kaplan–Meier analysis coupled with a log-rank test to assess possible associations of GULP1 expression with patient outcomes. However, there were no significant differences in progression-free survival in patients with non-muscle-invasive disease between GULP1-low and GULP1-high tumors (0 vs. 1+/2+/3+: *P* = 0.396 (Figure 5b); 0/1+ vs. 2+/3+: *P* = 0.285), as well as in those with muscle-invasive disease between GULP1-low and GULP1-high tumors (0 vs. 1+/2+/3+: *P* = 0.744 (Figure 5c); 0/1+ vs. 2+/3+: *P* = 0.193).

In another set of TMA consisting of muscle-invasive bladder cancer specimens from those who had subsequently undergone CDDP-based neoadjuvant chemotherapy, GULP1 was positive in 32 (74%) of 43 cases (1+: 17 (40%); 2+: 14 (33%); 3+: 1 (2%)). When GULP 0/1+ vs. 2+/3+ cases were compared, low expression was seen in 8 (47%) responders and in 20 (77%) non-responders (Table 2). Thus, low GULP1 expression was significantly associated with chemoresistance (*P* = 0.044).

In our previous study [13], AR was stained in the 43 bladder tumors included in the second set of TMA, showing immunoreactivity in 13 (30%) cases. GULP1 was positive in 24 (80%) of 30 AR-negative cases vs. 8 (62%) of 13 AR-positive cases. Thus, the rate of GULP1 positivity was higher in AR-negative tumors than in AR-positive tumors. However, the association of GULP1 and AR expression was not statistically significant (*P* = 0.262).

## 3. Discussion

Although resistance to CDDP-based chemotherapy is not uncommonly seen in patients with urothelial cancer, its underlying mechanisms are not fully understood. Meanwhile, AR activation in bladder cancer cells has been implicated to be associated with chemoresistance [12,13,14,15,21,22,23,24]. In the present study, we further investigated the role of GULP1, as a downstream target of AR, in CDDP resistance, using bladder cancer cell lines as well as surgical specimens.

Caenorhabditis elegans CED-6 and its human homologue GULP1, as adapter proteins, play an important role in phagocytosis [18,25]. Specifically, CED-6/GULP1 was shown to interact with phagocytic receptor CED-1 and other transmembrane receptor proteins, such as stabilin-1, and thereby engulf apoptotic cells. GULP1 was also shown to induce the rearrangement of the actin cytoskeleton via MAPK activation [26]. By contrast, its precise function in cancer progression remains largely unknown. Nonetheless, in ovarian cancer cells, GULP1 showed inhibitory effects on their proliferation while inducing phospho-SMAD3 [27] or reducing phosphorylation of AKT/PDK1 and MAPK [28]. In bladder cancer specimens, loss of immunoreactivity for GULP1 was more frequently seen in muscle-invasive tumors (85.8%) than in non-muscle-invasive tumors (39.0%) [29]. The reduced expression of GULP1 is also associated with poorer patient outcomes in The Cancer Genome Atlas dataset [30]. In bladder cancer lines, overexpression or silencing of GULP1, as a tumor suppressor, resulted in reduction or induction, respectively, of cell proliferation, migration, and invasion [29]. Moreover, GULP1 was suggested to involve CDDP resistance by showing an increase in cell viability and a decrease in apoptosis in a GULP1-silenced bladder cancer T24 line cultured in the presence of 20 µM CDDP, as well as a decrease in GULP1 expression in a CDDP-resistant T24 subline [29]. In addition, the levels of *GULP1* mRNA expression were found to be low (or undetectable) in all nine bladder tumors from patients who did not respond to CDDP therapy but were relatively high in two of six responders [29]. It is also worth mentioning that phagocytosis has been implicated in chemoresistance. For example, CDDP treatment in lung cancer cells was shown to induce CD47 expression, leading to a reduction in the phagocytic activity of co-cultured macrophages, while the CD47 blockade enhanced the cytotoxic effect of CDDP [31].

Here, we confirmed some of the previous findings [29] in two other bladder cancer lines and transurethral resection specimens. Specifically, GULP1 knockdown via stable expression of its shRNA resulted in a significant reduction in the cytotoxicity of CDDP, along with a significant decrease in apoptosis and a significant increase in G2/M population, both of which were altered by CDDP. Immunohistochemistry in surgical specimens from patients who had subsequently undergone CDDP-based neoadjuvant chemotherapy further showed significantly higher levels of GULP1 expression in those from responders (*n* = 17) compared with non-responders (*n* = 26). We additionally demonstrated that the levels of GULP expression were significantly or marginally lower in urothelial neoplasms (vs. non-neoplastic urothelial tissues) and high-grade or muscle-invasive tumors (vs. lower grade or non-muscle-invasive tumors), while we failed to show a strong association between the expression level of GULP1 and the prognosis of the patients who had never received CDDP (prior to tumor recurrence or disease progression). These results indicate that GULP1 plays a critical role in increasing sensitivity to CDDP. Inconsistent with previous observations, however, we found no considerable impact of GULP1 on the proliferation/migration/invasion or apoptosis in 647V cells cultured in the absence of CDDP.

As mentioned above, it remains unclear how AR signals modulate chemosensitivity. Based on our DNA microarray analysis data [16], we expected that GULP1 was a downstream target of AR. We herein demonstrated an inverse relationship between AR activity and GULP1 expression. Specifically, AR overexpression/androgen treatment and AR knockdown/anti-androgen treatment reduced and induced, respectively, the levels of GULP1 expression in bladder cancer cells. Remarkably, a ChIP assay revealed direct interactions of AR with GULP1 at its promoter region, further indicating direct regulation of GULP1 expression by AR. It is thus likely that GULP1 represents a key downstream effector of AR signaling in modulating CDDP sensitivity in bladder cancer. Meanwhile, several molecules/pathways have been suggested to be downstream targets of GULP1. In particular, it has been documented that GULP1 activates SMAD3 [27] or inactivates AKT/PDK1 and MAPK [28] in ovarian cancer cells, while it inhibits the nuclear translocation of NRF2 and subsequently reduces the expression of HMOX1 in bladder cancer cells [29]. Interestingly, all of these potentially downstream of GULP1 have been linked to CDDP resistance [10,24,32,33,34,35]. Further studies are required for elucidating the molecular mechanisms responsible for AR/GULP1-mediated chemoresistance.

While AR activity must be considerably higher in men than in women, no studies have demonstrated significant sex-related differences in the prognosis of bladder cancer patients who undergo systemic chemotherapy. This may be due to a similar role of estrogen signaling in modulating chemosensitivity. Indeed, we recently showed an association between estrogen receptor (ER)-β activation and CDDP resistance in bladder cancer [36]. Accordingly, modulation of GULP1 expression by estrogen signaling may also need to be further investigated.

An inverse association between AR and GULP1 expression was seen in bladder cancer cell lines. In surgical specimens, we further demonstrated marginal or significant down-regulation of GULP1 expression in high-grade or muscle-invasive tumors. The levels of AR expression should therefore be elevated in high-grade/muscle-invasive bladder cancers compared with low-grade/non-muscle-invasive tumors. However, conflicting data exist regarding the expression of AR mRNA [19,37] and protein (as reviewed in [11,12]) in different grades/stages of bladder tumors. Specifically, in a meta-analysis of immunohistochemical studies, AR expression was shown to be rather significantly down-regulated in high-grade or muscle-invasive tumors [38]. These findings may also raise the possibility of GULP1 modulation by others, such as ER pathways, since significant increases in ERβ expression in high-grade/muscle-invasive bladder cancers have been documented [38,39,40].

## 4. Materials and Methods

### 4.1. Antibodies and Chemicals

We obtained anti-AR (N-20), anti-GULP1 (E-4), and anti-GAPDH (6c5) antibodies from Santa Cruz Biotechnology (Dallas, TX, USA). DHT, HF, and CDDP were from Sigma-Aldrich (St. Louis, MO, USA).

### 4.2. Cell Lines

Human urothelial carcinoma cell lines, 5637, 647V, UMUC3, and TCCSUP, were originally obtained from the American Type Culture Collection (Manassas, VA, USA) and recently authenticated by the institutional core facility. Sublines stably expressing human wild-type AR (or vector only) or AR-shRNA (or control-shRNA), including 5637-AR [41], 647V-AR [42], and UMUC3-AR-shRNA [41], were established in our previous studies. Similarly, GULP1-shRNA (TRCN0000029057; MISSION^®®^ shRNA Bacterial Stock, Millipore Sigma, Burlington, MA, USA) was stably expressed in 647V and UMUC3 lines. These parental lines and sublines were maintained in DMEM (ThermoFisher, Waltham, MA, USA) supplemented with 10% fetal bovine serum (FBS), penicillin (50 U/mL), and streptomycin (50 μg/mL) at 37 °C in a humidified atmosphere of 5% CO_2_. The cells were then cultured in phenol red-free medium supplemented with 5% FBS at least 24 h before actual assays.

### 4.3. Western Blot

Equal amounts of proteins (30 µg) obtained from cell extracts were subjected to electrophoresis with 10% sodium dodecyl sulfate-polyacrylamide gel, which was transferred to a polyvinylidene difluoride membrane electronically. After blocking with 0.03–0.3% Blotting-Grade Blocker (BioRad, Hercules, CA, USA), the membrane was incubated with a primary antibody (i.e., AR (dilution 1:200), GULP1 (dilution 1:100), and GAPDH (dilution 1:1000)) at 4 °C overnight, followed by 1 h incubation with a HRP-conjugated secondary antibody (Cell Signaling Technology, Danvers, MA, USA) at room temperature. Chemiluminescent signals were generated by a Clarity western ECL substrate and detected by ChemiDOC™ MP (Bio-Rad).

### 4.4. ChIP

A ChIP assay was performed, using a Magna ChIP kit (Millipore Sigma) according to the manufacturer’s recommended protocol with minor modifications. UMUC3 cells were cross-linked with 1% formaldehyde for 10 min at room temperature. The cell lysates were sonicated in nuclear buffer (four 30-s pulses, output 3.0, duty cycle 30% in ice with 120 s rest between pulses; Branson Sonifier 450). Soluble chromatin was immunoprecipitated with an anti-AR antibody and normal mouse IgG (sc-2025, Santa Cruz Biochemistry) directly conjugated with Magnetic Protein A beads. Immuno-precipitated DNA was eluted and reverse cross-linked, and DNA was extracted and purified using a spin filter column. DNA samples were analyzed by PCR. We performed a bioinformatic search (LASAGNA-Search 2.0. Available online at https://biogrid-lasagna.engr.uconn.edu/lasagna_search/ [43]; accessed on 24 November 2020) for potential AR binding sites in the GULP1 promoter and found three target sites (see Figure 2). The sequences of the primers are as follows: Site 1 forward, CTGCGCCTATCACAACTCTATT; Site 1 reverse, GAAAGGGAGCAAGAAGGAGTATC; Site 2 forward, GGTCAGTAAGAATGGGCTGTT; Site 2 reverse, GGCCTTAATCTCTGGACTTTGT; Site 3 forward, CCAGAGATTAAGGCCGAGTTAAA; Site 3 reverse, AAACGTGCCGTCTTCACA. The PCR products electrophoresed on 1% agarose gel and stained with ethidium bromide were visualized using Gel Doc XR+ (Bio-Rad).

### 4.5. Cell Proliferation

We used the MTT assay to assess cell viability. Cells (5 × 10^3^/well) seeded in 96-well tissue culture plates were cultured for up to 96 h and then incubated with 0.5 mg/mL of MTT (3-(4,5-dimethylthiazol-2-yl)-2,5diphenyltetrazolium bromide, Sigma-Aldrich) for 3 h at 37 °C. MTT was dissolved by dimethyl sulfoxide, and the absorbance at 570 nm was measured. IC50 was calculated using the web-based tool (AAT Bioquest IC50 Calculator. Available online at https://www.aatbio.com/tools/ic50-calculator; accessed on 12 July 2021).

### 4.6. Apoptosis and Cell Cycle Analysis

The TUNEL assay was conducted on cell-burdening coverslips, using the DeadEnd Fluorometric TUNEL system (Promega, Madison, WI, USA), followed by counterstaining for DNA with 4′,6-diamidino-2-phenylindole. The apoptotic index was determined in the cells visualized by the fluorescence microscopy.

For cell cycle phase quantification, the Cell Cycle Assay Cell-Clock™ (Biocolor, Carrickfergus, UK) was used according to the manufacturer’s recommended protocol. Live cells exhibit color changes associated with cell cycle phases. Data were analyzed using ImageJ version 1.53 (National Institutes of Health, Bethesda, MD, USA).

### 4.7. Cell Migration

A scratch wound-healing assay was adapted to evaluate the ability of cell migration. Cells at a density of ≥90% confluence in 12-well tissue culture plates were scratched manually with a sterile 200 μL plastic pipette tip. The wounded monolayers of the cells were allowed to heal in serum-free medium for 24 h, and the width of the wound area was monitored with an inverted microscope. The normalized cell-free area in photographed pictures (24 h/0 h) was quantitated using ImageJ.

### 4.8. Cell Invasion

Cell invasiveness was determined using a Matrigel-coated transwell chamber (6.5 mm diameter polycarbonate filter with 8 µm pore size, Corning Inc., Corning, NY, USA). Cells (1 × 10^5^) in 100 μL serum-free medium were added to the upper chamber, whereas 600 µL medium containing 10% FBS was added to the lower chamber. After incubation for 24 h, invaded cells were fixed, stained with 0.1% crystal violet, and counted.

### 4.9. Immunohistochemistry

Two sets of TMA consisting of retrieved bladder tissue specimens obtained by transurethral surgery performed at the Johns Hopkins Hospital were previously constructed [13,39]. The first set consisted of 129 cases of urothelial neoplasm with various tumor grades/stages, along with normal-appearing urothelial tissues from the same patients. In these patients, CDDP was not used prior to tumor recurrence or disease progression. The second set consisted of 43 cases of high-grade muscle-invasive urothelial carcinomas that had subsequently received CDDP-based neoadjuvant chemotherapy prior to radical cystectomy, including 17 responders and 26 non-responders, as defined previously [13,44]. None of the patients included in these sets of TMA had received therapy with radiation or anti-cancer drugs prior to the collection of the tissues.

Immunohistochemical staining was performed on the 5 µm sections using a primary antibody to GULP1 (dilution 1:50), as we described previously [9,13,24]. All stains were manually quantified by a single pathologist (H.M.) who was blinded to sample identity. The final scores (range: 0–12) calculated by multiplying the percentage of immunoreactive cells (0% = 0; 1–10% = 1; 11–50% = 2; 51–80% = 3; 81–100% = 4) by staining intensity (negative = 0; weak = 1; moderate = 2; strong = 3) were considered negative (0; score < 2), weakly positive (1+; 2 ≤ score ≤ 4), moderately positive (2+; 4 < score ≤ 8), and strongly positive (3+; score > 8).

### 4.10. Statistical Analysis

Student’s *t*-test was used to compare numerical data. Fisher’s exact test or chi-square test was used to evaluate the associations between categorized variables. Survival rates in patients were calculated by the Kaplan–Meier method and comparison was made by log-rank test. All statistical analyses were performed using Prism version 5 (GraphPad, San Diego, CA, USA) and EZR software [45], a graphical user interface for R version 4.0.2 (The R Foundation for Statistical Computing, Vienna, Austria). *P* values less than 0.05 were considered to be statistically significant.

## 5. Conclusions

We identified GULP1 as a key downstream effector of AR in modulating CDDP sensitivity in bladder cancer. While GULP1 activators are not currently available, our data further support the notion that concurrent anti-androgen therapy has the potential of being a means of chemosensitization, especially in male patients with AR-positive bladder tumor. In addition, the expression status of GULP1, along with that of AR, may serve as a predictor of chemosensitivity in patients with bladder cancer.

## Figures and Tables

**Table 1:** Correlations between GULP1 expression in bladder cancers and their clinicopathologic features.

	n	Expression Levels				*P* Value	
		Negative	Positive			0 vs. 1+/2+/3+	0/1+ vs. 2+/3+
		0	1+	2+	3+		
Tissues						0.005	0.006
Non-neoplastic urothelium	89	9 (10%)	41 (46%)	33 (37%)	6 (7%)		
Urothelial neoplasm	129	33 (26%)	63 (49%)	30 (23%)	3 (2%)		
Tumor Grade						0.062	1.000
PUNLMP + LG	50	8 (16%)	29 (58%)	13 (26%)	0 (0%)		
HG	79	25 (32%)	34 (43%)	17 (22%)	3 (4%)		
Pathologic Stage						0.007	0.014
Non-muscle-invasive	78	13 (17%)	39 (50%)	25 (32%)	1 (1%)		
Muscle-invasive	51	20 (39%)	24 (47%)	5 (10%)	2 (4%)		
Lymph Node Involvement						0.331	1.000
pN0	36	13 (36%)	17 (47%)	4 (11%)	2 (6%)		
pN1-3	13	7 (54%)	4 (31%)	2 (15%)	0 (0%)		

PUNLMP, papillary urothelial neoplasm of low malignant potential; LG, low-grade urothelial carcinoma; HG, high-grade urothelial carcinoma.

**Table 2:** GULP1 expression in bladder cancer and response to chemotherapy.

		GULP1 Expression		*P* Value
		0/1+	2+/3+	
Responders	17	8 (47%)	9 (53%)	0.044
Non-responders	26	20 (77%)	6 (23%)	

## Data Availability

The data presented in this study are available on request from the corresponding author but are not publicly available due to privacy and/or ethical restrictions.
